# The impact of digital technology in care homes on unplanned secondary care usage and associated costs

**DOI:** 10.1093/ageing/afae004

**Published:** 2024-02-13

**Authors:** Alex Garner, Jen Lewis, Simon Dixon, Nancy Preston, Camila C S Caiado, Barbara Hanratty, Monica Jones, Jo Knight, Suzanne M Mason

**Affiliations:** Lancaster Medical School, Lancaster University, Lancaster, UK; School of Health and Related Research, The University of Sheffield, Sheffield, UK; School of Health and Related Research, The University of Sheffield, Sheffield, UK; Division of Health Research, Lancaster University, Lancaster, UK; Department of Mathematical Sciences, Durham University, Durham, UK; Population Health Sciences Institute, Newcastle University, Newcastle Upon Tyne, UK; Faculty of Health and Medicine, University of Leeds, Leeds, UK; Lancaster Medical School, Lancaster University, Lancaster, UK; School of Health and Related Research, The University of Sheffield, Sheffield, UK

**Keywords:** telehealth, emergency medicine, long-term care, care homes, routinely collected data, older people

## Abstract

**Background:**

A substantial number of Emergency Department (ED) attendances by care home residents are potentially avoidable. *Health Call Digital Care Homes* is an app-based technology that aims to streamline residents’ care by recording their observations such as vital parameters electronically. Observations are triaged by remote clinical staff. This study assessed the effectiveness of the Health Call technology to reduce unplanned secondary care usage and associated costs.

**Methods:**

A retrospective analysis of health outcomes and economic impact based on an intervention. The study involved 118 care homes across the North East of UK from 2018 to 2021. Routinely collected NHS secondary care data from County Durham and Darlington NHS Foundation Trust was linked with data from the Health Call app. Three outcomes were modelled monthly using Generalised Linear Mixed Models: counts of emergency attendances, emergency admissions and length of stay of emergency admissions. A similar approach was taken for costs. The impact of Health Call was tested on each outcome using the models.

**Findings:**

Data from 8,702 residents were used in the analysis. Results show Health Call reduces the number of emergency attendances by 11% [6–15%], emergency admissions by 25% [20–39%] and length of stay by 11% [3–18%] (with an additional month-by-month decrease of 28% [24–34%]). The cost analysis found a cost reduction of £57 per resident in 2018, increasing to £113 in 2021.

**Interpretation:**

The introduction of a digital technology, such as Health Call, could significantly reduce contacts with and costs resulting from unplanned secondary care usage by care home residents.

## Key Points

- We provide a retrospective analysis of the Health Call app in care homes using routinely collected data.
- Care home staff can upload observations of residents using Health Call to be triaged by a clinician.
- Health Call reduces monthly emergency attendances and admissions for care home residents.
- Residents using Health Call experience shorter emergency hospital stays.
- Costs to the health system are reduced, with an increasing trend in savings over the study period.

## Introduction

There are around 17,700 care homes in UK with around 430,000 residents. Most residents are over 80 years old with varying levels of complex healthcare needs. Hospital attendances and admissions can be hazardous for residents, with high rates of hospital-acquired infections, increased confusion and falls.

Generally, older patients prefer to be treated at their normal place of residence, but current NHS service configurations frequently struggle to achieve this. One aspect of the problem is high rates of Emergency Department (ED) attendance and hospital admissions. The NHS Long Term Plan [1] commits to better healthcare provision for care home residents.

The potential scope for reducing these, and the associated patient benefits and cost savings have been explored [2], and ready access to advice from healthcare professionals was cited as fundamental to delivering these reductions. Digital technology may be a scalable and cost-effective method to support timely advice, shared decision making and deliver closer working between agencies. However, evidence is needed to support these hypotheses along with an understanding of how to implement such tools to ensure appropriate uptake.

Health Call Solutions is a digital health initiative collaboratively run by seven NHS Foundation Trusts across North East RK and North Cumbria. One of the solutions provided is the Health Call Digital Care Homes Application (app), designed for use by staff in care homes [3]. A primary goal of the app is to reduce avoidable secondary care for the residents in the homes, through timely access to clinical advice (Box 1) [4, 5].

Health Call’s pilot area was County Durham and Darlington, a mixed rural/urban area in North East UK. We evaluated the effectiveness of the Health Call app by looking for changes in the utilisation of unplanned secondary care as well as associated costs to service providers for care home residents before and after Health Call is implemented in their care homes. This was done using a large, linked dataset of healthcare interactions within County Durham and Darlington NHS Foundation Trust (CDDFT) and data from the Health Call app.

 

Box 1: Description of the Health Call app system.

The app provides a structured method for seeking clinical advice for the management of care home residents who become unwell. Upon implementation of the system, the staff are trained to use it to record residents’ vital signs readings and other observations through a form on the app (see Supplementary Figure S1). A National Early Warning Score 2 (NEWS2) score is calculated from these observations on upload. The form also includes a section for free text describing a resident’s condition using a Situation, Background, Assessment, Recommendation (SBAR) approach, which is a structured form of communication used to enable information to be conveyed accurately. Information uploaded to the app is automatically fed into the resident’s Electronic Health Record (EHR) and a Single Point of Access (SPA) where clinical staff triage referrals with the context of the patients entire EHR and provide advice and next steps on the care for the residents. The SPA is monitored by clinical staff during working hours; outside of these hours, emergency presentations would require a more traditional approach.

The app replaces the traditional method of seeking advice through telephone calls with sometimes limited and incomplete information. It provides a faster response and advice for care home staff allowing staff and clinicians to work swiftly together on resident presentations, facilitating early identification of residents’ health problems.

## Methods

We utilised data from the Health Call app from its rollout in December 2018 until August 2021. Three care home datasets from Health Call covering resident enrolment, home enrolment and uploads on the app are linked to six routinely collected datasets from CDDFT, including ED, inpatient, outpatient and community nursing data. An additional dataset containing information on patients’ hospital discharges was also used. See Supplementary Table S1 for a description of each dataset. Recording practices for the data used in this study remained constant throughout the study period. Primary care and ambulance service data were not included.

### Linkage and cohort selection criteria

Each dataset used a pseudonymised NHS number as an individual identifier, meaning the same individual could be identified across all of the datasets. We defined the study cohort using registration data from the Health Call app. The registration data include dates when a care home resident was ‘activated’ and ‘deactivated’ from the system and their care home’s name. The activation date refers to when a resident was added onto the Health Call by the home. A resident is deactivated after they die or move away.

**Figure 1:**
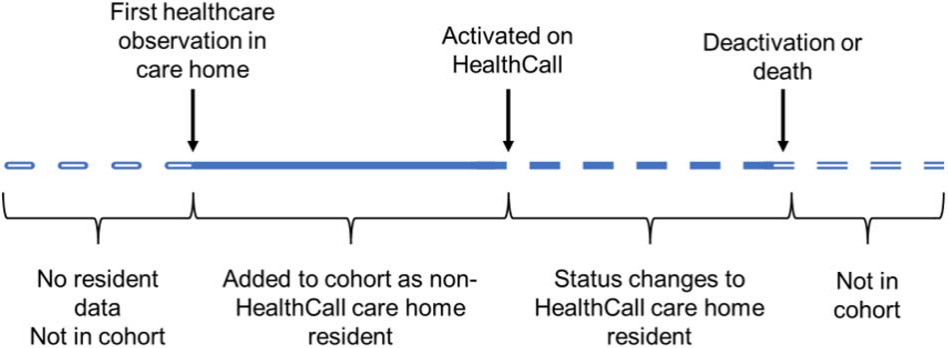
Diagram demonstrating residents’ transitions into the cohort and subsequent activation as a Health Call Resident.

A resident was included in the study cohort from the first date at which an observation from any of the datasets placed them in the home that they were activated in. From this date they are a ‘non-Health Call resident’ until their activation date. If they had no deactivation (or death) date, they were assumed to still be living in a home using Health Call at the end of the study period. Residents were removed from the cohort when there was a ‘deactivation date’ or an identified death date for the resident in any of the datasets. A typical resident timeline is shown in Figure 1. We also used observations to identify a small number of residents who were observed to be in the care homes that used the app, but were never ‘activated’ on the system who stayed as non-Health Call residents. Residents activated on Health Call who are not observed to have any healthcare interactions before their activation date are not included in the cohort.

### Primary investigation

We investigated three co-primary outcomes as potential indicators of change in unplanned secondary care usage. We hypothesised that the introduction of Health Call impacted the way residents and staff interacted with secondary care; related to ED usage and recovery from hospital stays. We investigated the following:

1. Monthly emergency attendances
2. Monthly emergency admissions
3. Length of stay of emergency stays

Further details of these outcomes can be found in the supplementary materials.

We also provide an economic evaluation to calculate change in costs to service providers due to the introduction of Health Call.

Service costs related to ambulance journeys to EDs, attendance at EDs, emergency inpatient stays and outpatient attendances were assigned a unit cost. These were summed to produce a total cost, at 2019/20 price levels, for each patient each month they were part of the study cohort.

ED and outpatient activity were costed using their associated healthcare resource group and National Reference costs for 2019/2020 [6]. For inpatient stays, National Reference costs for 2017/2018 were used [7] and inflated to 2019/2020 price levels using the NHS Cost Inflation Index [8]; these costs represent the most recent for which a cost per day can be derived. Visits to care homes by healthcare professionals were costed as either by a district nurse or community matron, with 1 hour of time being assigned to in-person visits and 15 minutes for other types of visit [8]. The full set of unit costs are shown in Table S3 (Supplementary Materials).

### Statistical modelling

We fitted a statistical model to each of the outcomes under investigation to understand typical patterns in these outcomes over time, and assess the impact of the introduction of the Health Call app. The gradual rollout of the app over the study period as well as the occurrence of the COVID-19 pandemic during the study period were key issues to address in the study design. These factors along with the retrospective nature of the study meant typical intervention evaluation methods, such as interrupted time series and difference-in-difference analysis would not suffice. They cannot account for issues such as the effect of the pandemic, many intervention time points and the dynamically changing cohort size.

To incorporate the different Health Call ‘activation’ times for each of the residents we fitted resident-level Generalised Linear Mixture Models (GLMM). We created baseline models that do not include Health Call as a predictor variable and compared to models including a binary variable indicating whether a resident is activated on the Health Call system. These models were fitted using the lme4 package in *R* [9].

The baseline GLMM was fit to resident-level outcomes, without accounting for Health Call. This model included a random intercept for care homes, and a nested random intercept for each resident. This structure allowed for variations between care homes and residents and reflects that each resident resides in only one care home. We used a Poisson model specification with a log-link function for the three patient outcome models.

The model contained five fixed effects variables, to account for typical seasonal patterns in outcomes as well as the impact of the COVID-19 pandemic, which occurred during the study period. The variables were a yearly harmonic pair (two sinusoidal curves to model cyclic fluctuations over the course of a year); month number (number of months passed of the study period); monthly CDDFT COVID-19 bed days (proxy for local COVID-19 prevalence to account for the impact of the pandemic); and pandemic wave (categorical variable to account for fluctuations in impact over the course of the pandemic). A mathematical description of the baseline model can be found in the Supplementary Materials.

For the economic outcome measure, costs were analysed in a similar fashion, but a two-part ‘hurdle’ model specification is used, given the nature and skew of these data; the best model based on a Cullen and Frey plot adopted logistic and gamma link-functions [10]. The logistic regression estimated the probability that a resident has zero costs in a given month, while the gamma regression estimated the costs contingent on a resident having non-zero costs. Cost per resident is then calculated based on the predictions of the two regressions. Implemented using the *glmmTMB* package in *R*.

The impact of Health Call was modelled as both an immediate step effect (binary main effect in the model) and an additional ongoing effect (as an interaction term between the binary Health Call variable and the linear month number variable). We conducted likelihood ratio tests (LRT) to assess the impact of including the step (baseline vs step model), then additionally the ongoing effect in the model (step model vs step and interaction term model). Due to the analysis of costs requiring a two-part model, step and ongoing impacts of Health Call were assessed for each of the associated regressions.

## Results

A total of 8,702 care home residents were identified and added to the cohort. The cohort selection criteria meant that the cohort grew over time as residents were identified based on appearances in observational datasets. The relative size of the group of non-Health Call residents depletes as residents are registered on Health Call over time. The overall cohort size and number of residents in each group can be seen in Supplementary Material Figure S2.

Of the 8,702 residents, 2,549 died within the study period. The median resident age was 85. Some residents were deactivated from the Health Call system for other reasons, for example, if they moved to a non-Health Call home. A summary of the characteristics of the cohort can be found in Table S2. Plots of raw outcomes can be found in Figures S3–S16.

**Figure 2:**
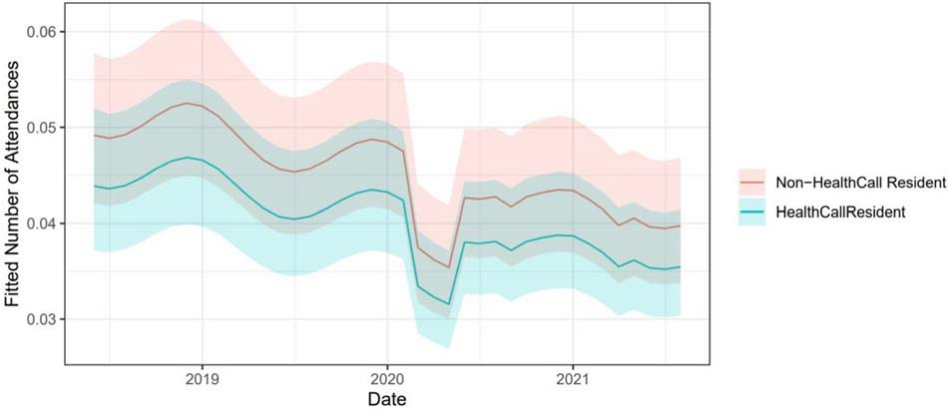
The expected number of ED attendances from the model over the study period for residents on the Health Call system and residents not on the Health Call system. Ribbons show 95% prediction intervals.

A demonstration of the model including the Health Call binary variable for monthly attendances fitted over the study period can be seen in Figure 2; this shows how the model varies over time and highlights the step change between the residents on the Health Call system (blue) and those that aren’t (red). An ongoing change was not included in this model since it was not found to be significant, hence the parallel lines. Results of the LRT, and the associated relative risks (derived from the coefficients) can be found in Table 1.

**Table 1:** Results table showing estimated relative risk for the Health Call step (main effect) and monthly change (linear interaction term) and associated statistics. *P*-values presented here are those of the LRT of including each outcome in the model

Outcome	Effect	Estimate (RR)	95% CI	LRT *P*-value
**Emergency attendances**	**Step**	0.892	0.846–0.941	<0.001
	**Ongoing**	1.003	0.999–1.008	0.1923
**Emergency admissions**	**Step**	0.751	0.708–0.795	<0.001
	**Ongoing**	1.015	1.009–1.021	1.000
**Length of stay**	**Step**	0.892	0.817–0.974	<0.001
	**Ongoing**	0.719	0.665–0.755	<0.001

Results discussed in this paper referring to the three outcomes are from the modelling. Raw results can be found in the supplementary materials. The number of ED attendances and admissions for residents on the Health Call system were typically 11 and 25% less than the non-Health Call residents. Length of emergency inpatient stays were reduced by 11%, with a slope indicating decreasing length of stay for Health Call residents of each month of the study reducing by 28% respective to the previous.

**Figure 3:**
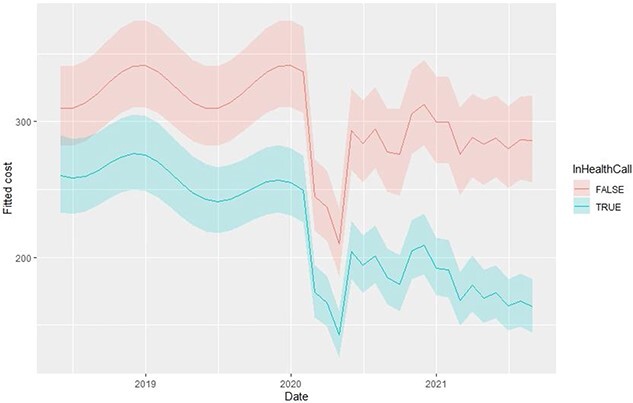
Predicted mean cost per resident of Health Call and non-Health Call homes over the study period.

Health Call was estimated to produce an immediate 27% reduction in the odds of a resident-month incurring zero costs; however, there was an estimated long-term trend of increasing odds per-month of zero-cost resident-months of 3% (Table 2). Health Call produced an immediate 24% reduction in non-zero costs. The longer-term trend in non-zero costs, while statistically significant, is small 0.03% (Table 2). Combined, these predictions show that there is an immediate decrease in the probability of a zero-cost and a reduction in non-zero costs, with the magnitude of the decreased costs becoming greater over time (Figure 3). The predicted values for each component part of the two-part model are shown in Figures S17 and S18. Predicted monthly costs per-resident for the four calendar years are shown in Table S4, and show a £57 reduction in cost per resident in 2018, increasing to a £113 reduction in 2021.

**Table 2:** Impact of Health Call on non-zero costs and the probability of zero monthly costs in the form of odds ratio (OR) for zero cost models and relative risks for the magnitude of costs model (RR). *P*-values presented here are those of the LRT of including this variable in the respective model

Outcome	Effect	Estimate	95% CI	LRT p-value
**Zero-cost (OR)**	**Step**	0.730	0.675–0.790	<0.001
	**Ongoing**	1.026	1.023–1.029	<0.001
**Magnitude of non-zero cost (RR)**	**Step**	0.762	0.716–0.810	<0.001
	**Ongoing**	1.003	1.000–1.006	0.024

## Discussion

This study suggests that the introduction of a digital technology intervention such as Health Call may significantly reduce contacts with and costs resulting from emergency care service use by care home residents. The modelling suggests an 11% reduction in estimated monthly number of ED attendances experienced by Health Call registered residents compared to non-Health Call registered residents. In addition, there was a 25% reduction in emergency hospital admissions further reducing impact on the hospital system. Health Call residents also experience 11% shorter emergency hospital stays, with an increasing reduction in stay over the study period of 29% compounded each month.

Improved communication between care home staff and the NHS, including NHS community nursing services, provides greater opportunity for joint decision making on the delivery of optimal care for residents, which we found led to a reduction in ED attendances and hospital admission. The associated qualitative study undertaken alongside this quantitative analysis [5] found that additional input from a multi-disciplinary team improves the confidence of care home staff by providing greater monitoring, and earlier identification of deterioration. This in turn may impact on the reduced length of hospital stay due to earlier detection and prompt management of illness.

The cost analysis indicates reduced health care costs for residents registered on the Health Call system, with the magnitude of this reduction increasing over time. This trend is driven by any given resident having an increasing probability of having zero costs over time. In the first year of operation, cost savings of £57 per resident-month were estimated, which equates to £247 million for the first year across UK, based on a care home population of 360,792 [4].

A key success of the paper is that the app was evaluated using routinely collected NHS data linked with observational data from the app. The methods presented attempt to minimise the impact of a challenging study period and gradual roll-out by modelling both intervention and non-intervention groups to calculate the impact of the intervention on outcomes. We demonstrate the research that can be done using a pragmatic approach to statistical analysis with routinely collected NHS data. The large amounts of data stored in NHS systems provide potential for more analyses such as this one.

As part of the NHS Long Term Plan, there was a promise to roll out Enhanced Health in Care Homes (EHCH), which highlights the use of technology for telehealth, remote monitoring and sharing of information to reduce uncoordinated care [11]. The Health Call system falls within this scope and this study demonstrates the impact of the technology on the healthcare system.

In a 2016 report, The Health Foundation stated that ED trips could be avoided by more data sharing between care homes and NHS services and use of clinical input in care homes [2]. Our results indicate that monitoring and administering of healthcare facilitated by the Health Call system could help address these issues. The report also highlights the challenges in accessing routinely collected data on care home residents. This study demonstrates the requirement of this linked data for appropriate evaluations and underlying the need to identify ways to make it more available such as the current NHS secure data environment program [12].

A number of digital interventions in care homes have been piloted in recent years, each with differing techniques to address the problems highlighted in the EHCH framework. The usage of telehealth has become particularly widespread since the start of the COVID-19 pandemic [13]. The Innovation Collaborative published a Rapid Review of remote monitoring technology in care homes [14]. The report identifies 19 remote monitoring technologies (including Health Call) used in the UK and Ireland, with 8 case studies and one published evaluation. There is a growing body of work on telehealth initiatives for older adults outside care homes [15]. The range of technologies becoming available highlights the need to evaluate their effectiveness using robust statistical methods, similar to those in this paper. Linkage of routinely collected hospital data with data collected through the usage of the technologies, as described here, provides a route for post-implementation evaluation of the technologies using only administrative data.

The study had a number of limitations. The data contained no timestamp of when a resident in the study first moved into long term care. Hospital discharge records were used to identify the date at which a resident was first observed to be in a care home. This identification method leads to a changing cohort size over time and class imbalances between the Health Call and non-Health Call residents. The study period was reduced prior to modelling to remove the months with the largest class imbalances. The model specification was used to account for the change in group sizes over time.

Residents were removed from the cohort when either deactivated from the Health Call system or they died. Since residents have generally been activated on the Health Call system before they are removed from the study (deactivation or death), a period of inactivity between actual and recorded deactivation could contribute to lower rates of healthcare utilisation, and therefore cost, for residents in the Health Call group.

Covariates in the model were limited by the available study data. Resident comorbidities and characteristics would be unreliable to identify using observational data. Linked home characteristics such as type of home and number of residents were also not included as they were found to not provide a significantly better fit to the model. Variation between residents and homes was instead captured by the hierarchical random intercept structure of the model.

This study was timely, as the onset of COVID-19 during the study period led to rapid uptake of Health Call. Our modelling aimed to disentangle the impact of Health Call from that of the COVID-19 pandemic on healthcare utilisation, by using a proxy for COVID prevalence and a pandemic wave variable. However, as the impact of the pandemic was immeasurable, results from this study may not reflect those that would have been observed during a non-pandemic period and type 1 error is possible.

For the costs analysis, two additional weaknesses are the lack of complete ambulance service data and the nature of the community contacts data. For ambulance data, only calls resulting in an ED attendance have been included in our cost estimates. For community contacts, length of contact was not available, and the profession of the health care worker was poorly defined, leading to imprecise allocation of unit costs to staff. However, these issues were consistent for both Health Call and non-Health Call residents, so confounding is likely to be minimal.

The estimated reduced costs reflect changes in the utilisation of NHS services. Not all changes across the health and social care system were included in our analysis, with the costs of the Health Call system and associated care home activities being the most prominent of those exclusions. While the cost of Health Call will be clear to Integrated Care Boards when purchasing the system, the potential costs to care homes are important to consider for successful implementation.

Our research provides key insights into how the introduction of a technology like Health Call impacts healthcare utilisation and cost outcomes. Future research could investigate the decision-making process in more detail, looking at decisions made from each individual upload from the app. This would allow for further investigation into the direct outcomes from the altered decision making provided by the app, to allow for a more detailed analysis of safety of decision-making.

The results shown in this paper are promising, but a definitive trial would help establish the true impact of the technology. Research over a larger area and longer time period, with more time before and after the intervention is introduced could improve reliability of results. The time period was limited by the data available. Randomisation of the Health Call roll-out over a wider area would be desirable to ensure findings are robust. Further research could also test technologies like Health Call in other settings such as mental health facilities.

## Supplementary Material

aa-23-1202-File002_afae004

## Data Access Statement:

Data was collected from CDDFT and stored in a Trusted Research Environment (TRE) managed by Durham University. Informed consent was not possible as the data were anonymised. The Trust shared anonymised data after undertaking a Data Privacy Impact Assessment and a Data Transfer Agreement. Data supporting this study is not publicly available due to ethical considerations around accessing linked patient-level healthcare data. Please contact the main author for more information (a.garner2@lancaster.ac.uk).
