# Supplementary material for: The impact of digital technology in care homes on unplanned secondary care usage and associated costs
Source: Age Ageing. 2024 Feb 13;53(2):afae004. doi: 10.1093/ageing/afae004 (PMC10861323; doi:10.1093/ageing/afae004)
Supplement: aa-23-1202-File002_afae004 [file aa-23-1202-file002_afae004.docx]

**Supplementary Material**

**The Impact of Digital Technology in Care Homes on Unplanned Secondary Care Usage and Associated Costs**

Contents

[Data Description 1](#_Toc153913642)

[Health Call Information 3](#_Toc153913643)

[Outcome Definitions 3](#_Toc153913644)

[Model Specification 4](#_Toc153913645)

[Cohort Information 4](#_Toc153913646)

[Raw Results 5](#_Toc153913647)

[Additional Economic Analysis Information 10](#_Toc153913648)

### Data Description

Table S 1: A description of each of the datasets that was available for use in this analysis.

| Healthcare Events Datasets | Description |
| --- | --- |
| ED | Details of attendances at 5 ED departments covered by CDDFT including the two major acute hospitals: Darlington Memorial Hospital and University Hospital of North Durham. Date and location of attendance is included, along with details of investigative procedures carried out on the patient and diagnosis codes. |
| Inpatient | Details of inpatient spells in the CDDFT hospitals. Dates for duration of overall stay and ward episodes within the stay are included. ICD-10 (International Statistical Classification of Diseases and Related Health Problems 10^th^ Revision) codes detailing diagnosis and comorbidities. |
| Inpatient Observations | Early Warning Scores of inpatients during their hospital stay (no constituent vital sign observations). Includes ward code of stay, date and time observation was made. |
| Outpatient | Details of outpatient appointments. Includes date and duration of interaction. Includes specialty of staff responsible for the patient. |
| Ward Episodes | Details of patient ward episodes during their hospital stays. Includes the ward code of the episode. |
| Community | Details of community appointments and callouts in the County Durham and Darlington area. Date and location type (conducted at patient’s home, in community hospital etc) are included, along with care plan details indicating the reason for the interaction. |
| Health Call | EWS observations of care home residents logged on the Health Call app by carers. Contains the separate observations that contribute towards calculating an EWS score and the time the observations were taken. |
| Additional Data Sets | |
| Discharges | Summary dataset of hospital visits, including number of hospital visits and dates of discharge from hospital. Also includes care home (if applicable) of patient mined from hospital records, and date of death (if applicable) contained in hospital records of the patient. Used as a lookup table for patient death dates. |
| Health Call Referrals | Dates of activation and deactivation of care home residents on the Health Call system. Activation dates refer to the date they are first put onto the Health Call system, may be when Health Call first goes live in the care home, or when the resident first moves to the care home. Conversely, deactivation dates may refer to the date a resident leaves the care home (moves care home or goes back to own accommodation) or dies. The data identifies the most recent care home each resident has been assigned to, providing an indicator of each resident’s care home. |
| Health Call Implementation | Dates each Health Call care home ‘went live’ and implemented Health Call. This is the only non-patient level dataset. |
|  |  |

###
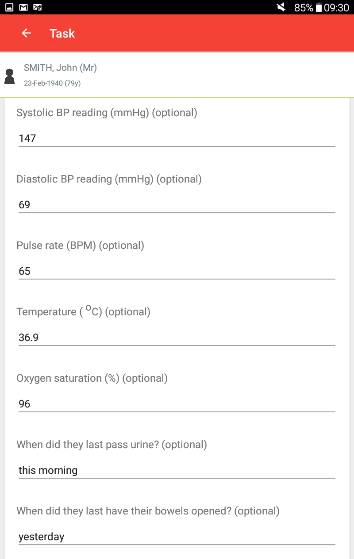

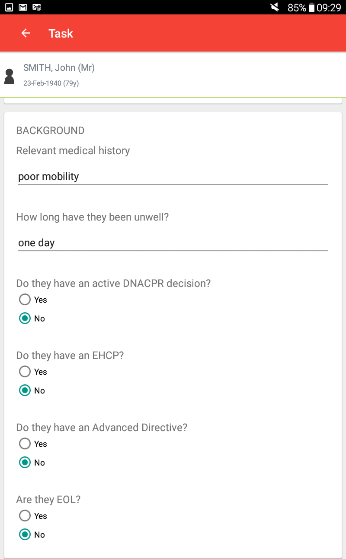

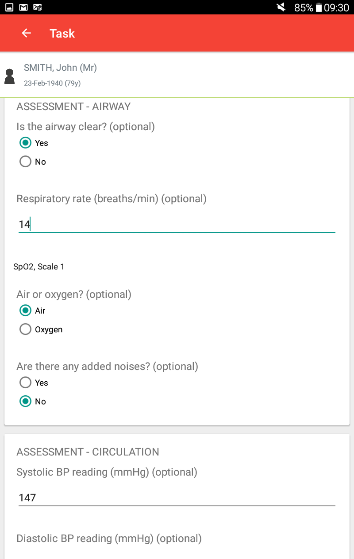
Health Call Information


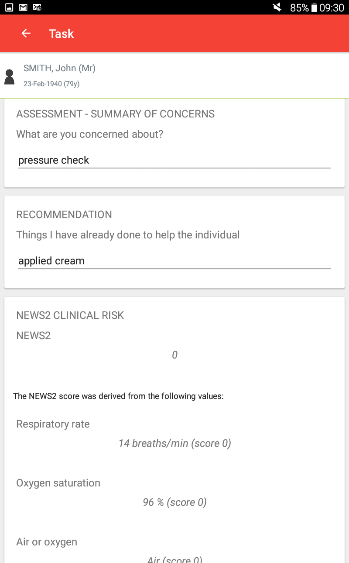

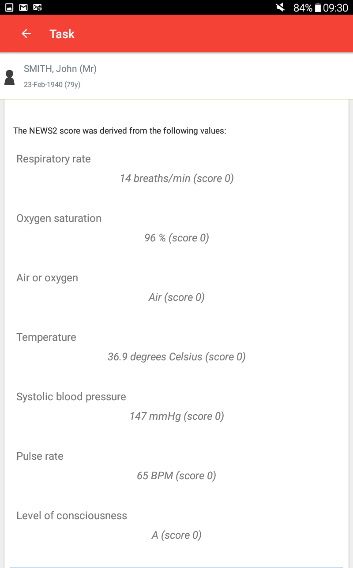

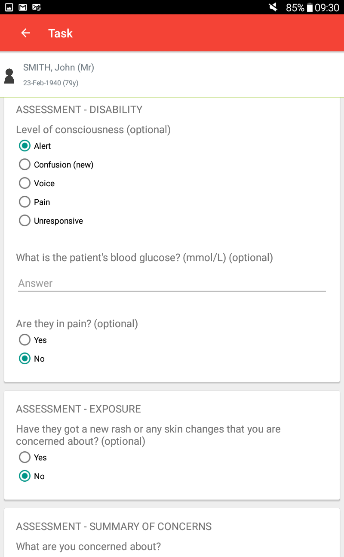


Figure S 1: Screenshots of the Health Call app. They show the form for inputting resident status and readings.

Resident details are input through the form in the app. Figure S 1 is an example of the form that will be filled out by the care home staff. The form is in the Situation, Background, Assessment, Recommendation structure. The information in this form is then sent to the clinicians at the Single Point of Access (SPA). The clinicians review the observations and action as appropriate. The resident’s electronic patient record is also updated.

### Outcome Definitions

Outcomes are modelled on a disaggregated resident-basis. The first two model estimates are an expected count for one resident in a month. The third is the length of stay. The outcomes are counted when the patient is classed as a care home resident at the point in time of their healthcare interaction (see main paper *Linkage and Cohort Selection Criteria*).

1. Monthly emergency attendances. Calculated from the ED data. Raw numbers are the count of attendances each resident experienced in each month of the study period.
2. Monthly emergency admissions. Calculated from the ED data. Raw numbers are the count of admissions to hospital from the ED that each resident experienced in each month of the study period.
3. Emergency length of stay. Calculated from the inpatient data, only including stays classed as emergency. In this outcome, residents can have multiple stays each month. Raw data is length of stay for each stay, grouped by admission month. Modelling is essentially expected length of stay for stays beginning in a given month. Lack of stays does not contribute to the model in this case (like a count of zero attendances in a month would).

### Model Specification

Let $y$ be the outcome variable of the investigation. $i$, $\left( i=1\ldots n \right)$ corresponds to a resident from care home $j$, $\left( j=1\ldots g \right)$.

Outcome $Y$ is the monthly count of events for each resident (this will vary dependent on the outcome we are modelling), we assume that these counts follow a Poisson distribution. In the length of stay outcome, it is a count of days for each stay, rather than events in a given timeframe. $\mu$ is the estimated value of the distribution. $h$ is the link function between the linear predictor and outcome $y$, decided depending on the distribution of $Y$.

The proposed baseline model will take the form;

$$Y_{ijk}\sim Poisson\left( y_{ijk};\lambda_{ijk} \right)$$

$$\eta_{ijk}=ln \left( \lambda_{ijk} \right) =\beta_{0}+\beta_{1}x_{1ijk}+\beta_{2}x_{2ijk}+\ldots+b_{j}+c_{ij}+\epsilon_{ijk}$$

$$b\sim Normal\left( 0,\tau_{b}^{2}I_{g} \right)$$

$$c_{j}\sim Normal\left( 0,\tau_{c}^{2}I_{n} \right)$$

$$\epsilon_{ijk}\sim Normal\left( 0,\sigma_{ijk} \right) ,$$

where $\eta$ is the linear predictor, $\beta$ are the regression coefficients. $x_{pijk}$ is the $k$th observation of the $p$th variable of individual $i$ from care home $j$. $b_{0}$ corresponds to variation on a care home level, and $c_{0}$ corresponds to variation on an individual level. The individual level random intercept is nested within the care home level since each individual lives in only one care home.

### Cohort Information

The number of residents in the cohort increased over the study period. At the start of the period most residents were non-Health Call residents. There was a large spike in uptake of the app during early 2020, coinciding with the start of the COVID-19 pandemic. Beyond this timepoint most of the residents in the cohort were Health Call residents. The roll-out can be seen in *Figure S2.*


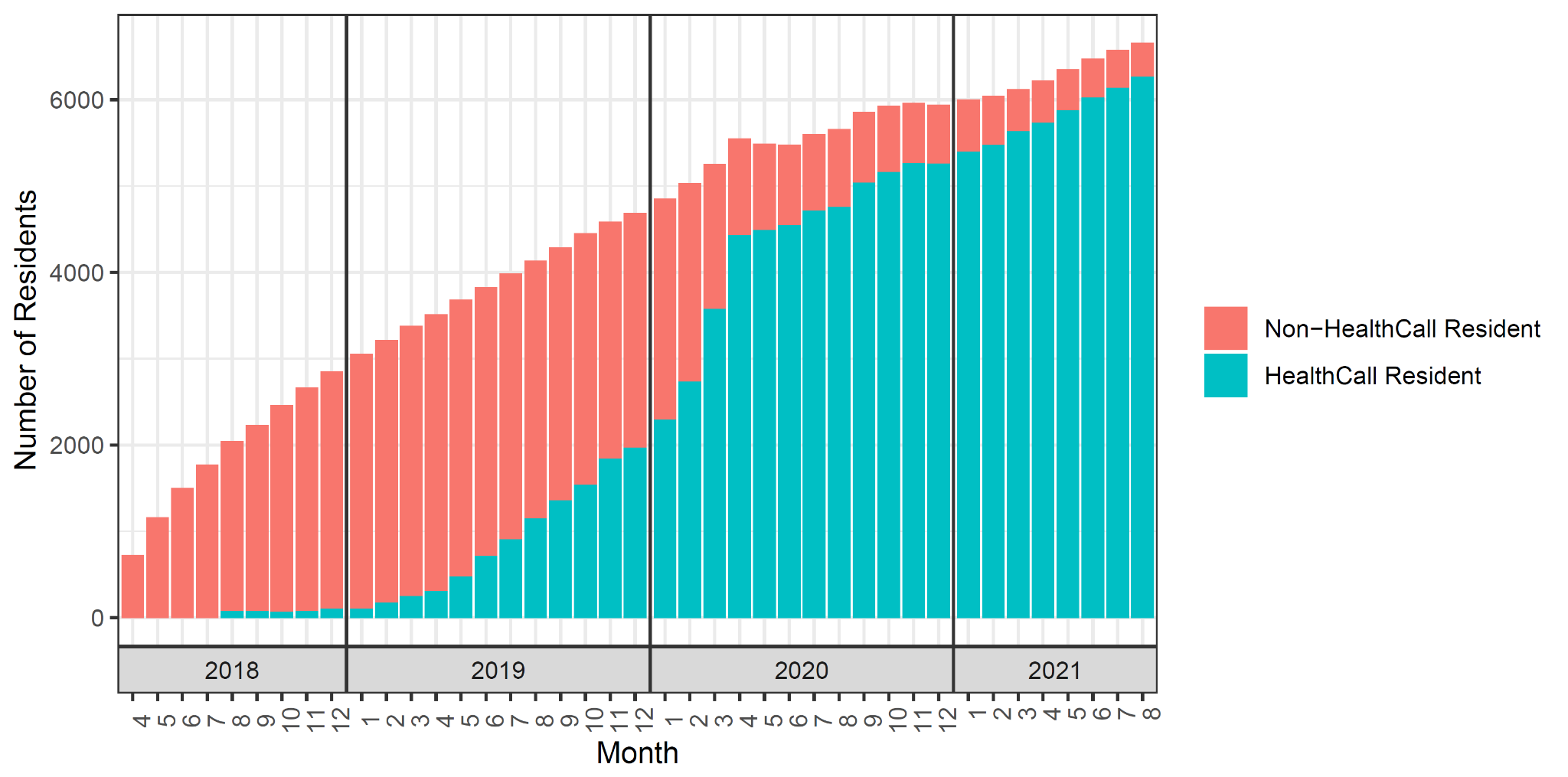


Figure S 2: Number of residents in the cohort in each month of the study. The colours separate the groups of non-Health Call and Health Call residents.

Characteristics of the identified residents can be found in *Table S2*. Resident ages were classed as the oldest observed age from any of the available datasets.

Table S 2: Characteristics of the cohort of care home residents included in the study.

|  | **Median** | | **IQR** | |
| --- | --- | --- | --- | --- |
| Age * | 85 | | 79-90 | |
| Number of Observations | 58 | | 29-109 | |
| Months in cohort | 19 | | 11-31 | |
| Months as non-Health Call resident | 13 | | 5-19 | |
| Months as Health Call resident | 14 | | 6-18 | |
|  | **Male** | | **Female** | |
| Gender | 3,086 (35%) | | 5,616 (65%) | |
|  | **True** | | **False** | |
| Died (within the study period) | 2,549 (29%) | | 6,153 (71%) | |
|  | 0 | 1-2 | 3-4 | ≥5 |
| Charlson Comorbidity Index ** | 324 (8%) | 2,111 (52%) | 1,292 (32%) | 324 (8%) |

***Table S2 Legend****: * We do not have age information for 1,394 of the residents. ** We could not calculate a Charlson Comorbidity Index for 4,671 residents due to them not having registered ICD-10 codes from their inpatient stay. Percentages are of those calculated.*

### Raw Results

We model expected counts of ED attendances and admissions on a resident-level. Looking at total numbers (Figures S3 and S5) and numbers broken down by resident’s Health Call status (*Figures S4 and S6*), we can see the impact of the changing cohort size. The changing cohort size can be seen in *Figure S2*.

The length of stay analysis is essentially mean length of stay for stays beginning in a given month. Therefore, we have provided number of stays for each month (*Figures S11* and *S12*), and number of bed days for each month (*Figures S13* and *S14*). Then combined to find each month’s overall mean (*Figure S15*) and broken down by Health Call status (*Figure S16*).

#### Monthly Emergency Attendances


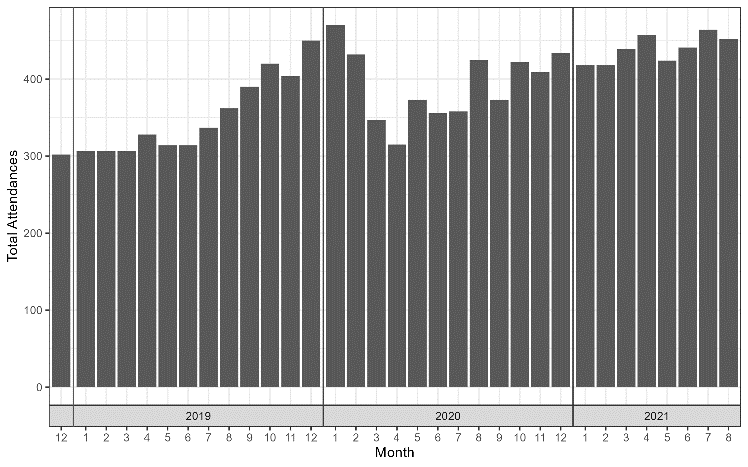


Figure S 3: Total number of ED attendances each month during the study period for residents in the cohort.


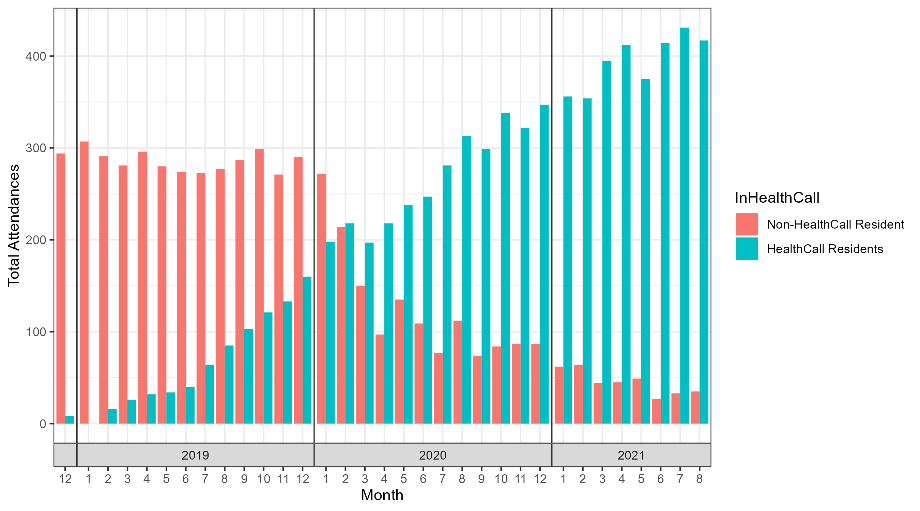


Figure S 4: Total number of ED attendances each month during the study period for residents in the cohort, separated by residents' Health Call statuses.


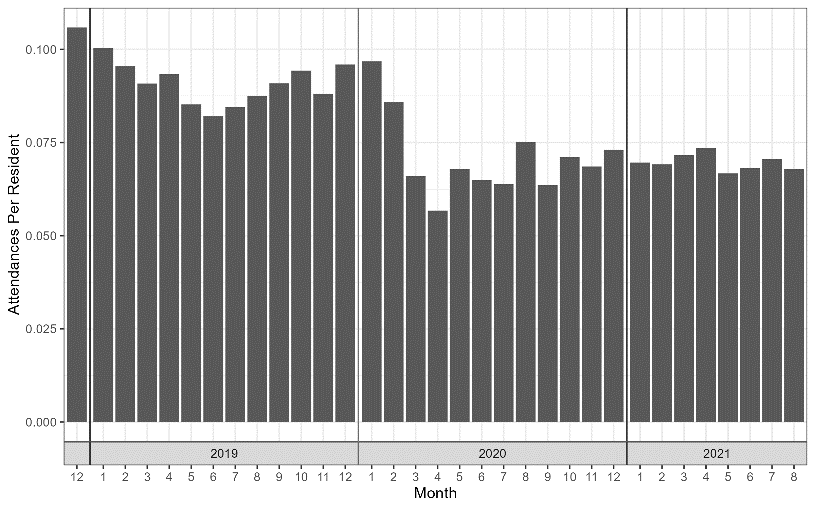


Figure S 5: Number of ED attendances per resident for each month of the study period.


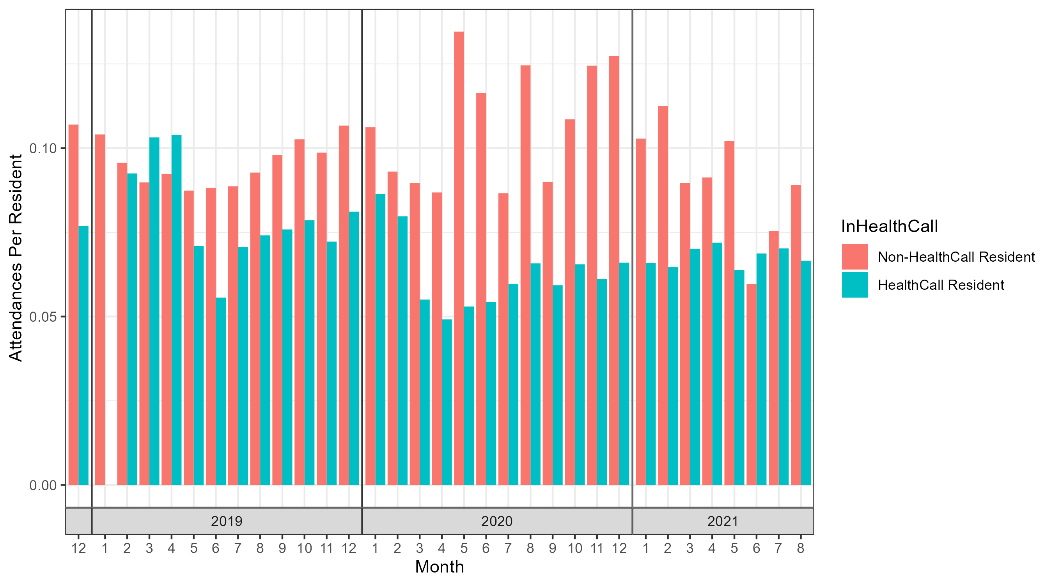


Figure S 6: Number of ED attendances per resident for each month of the study period, calculated separately for residents on the Health Call system at that point and those who aren’t.

#### Monthly Emergency Admissions


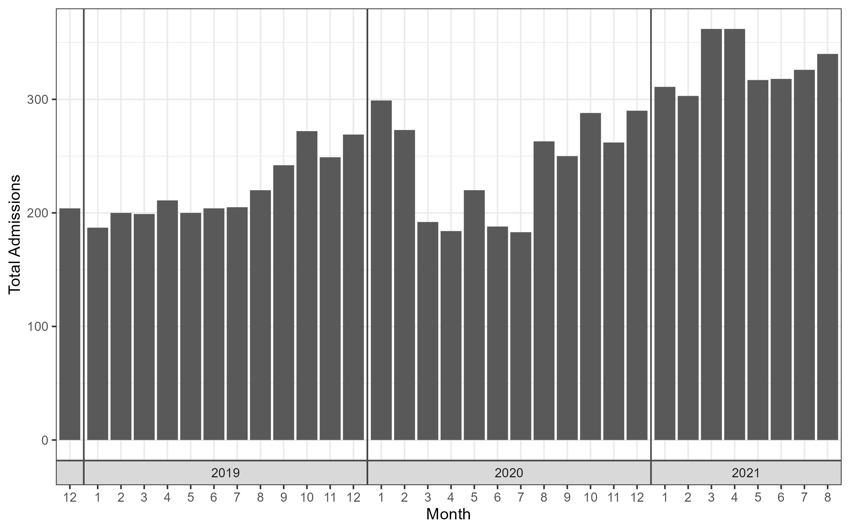


Figure S 7: Total number of emergency admissions each month during the study period for residents in the cohort.


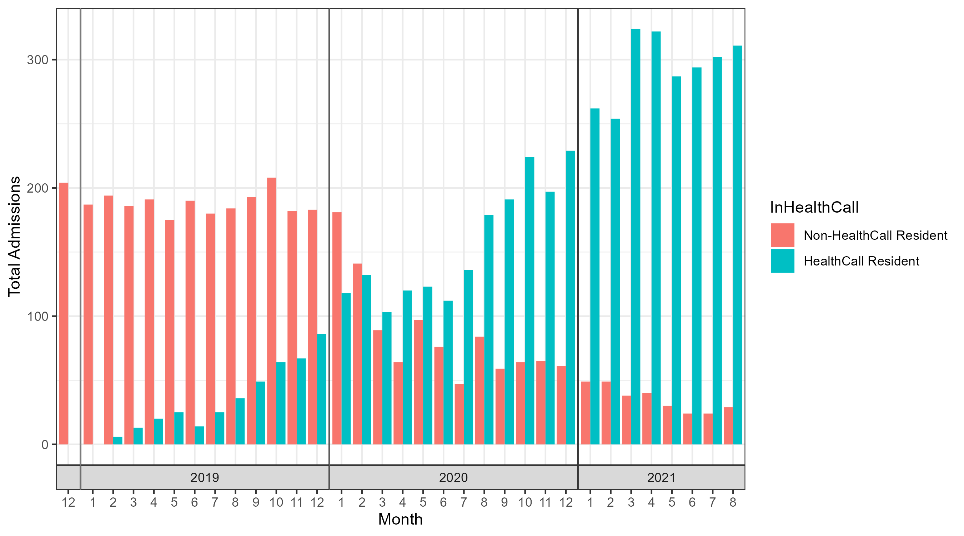


Figure S 8: Total number of emergency admissions each month during the study period for residents in the cohort, separated by residents' Health Call statuses.


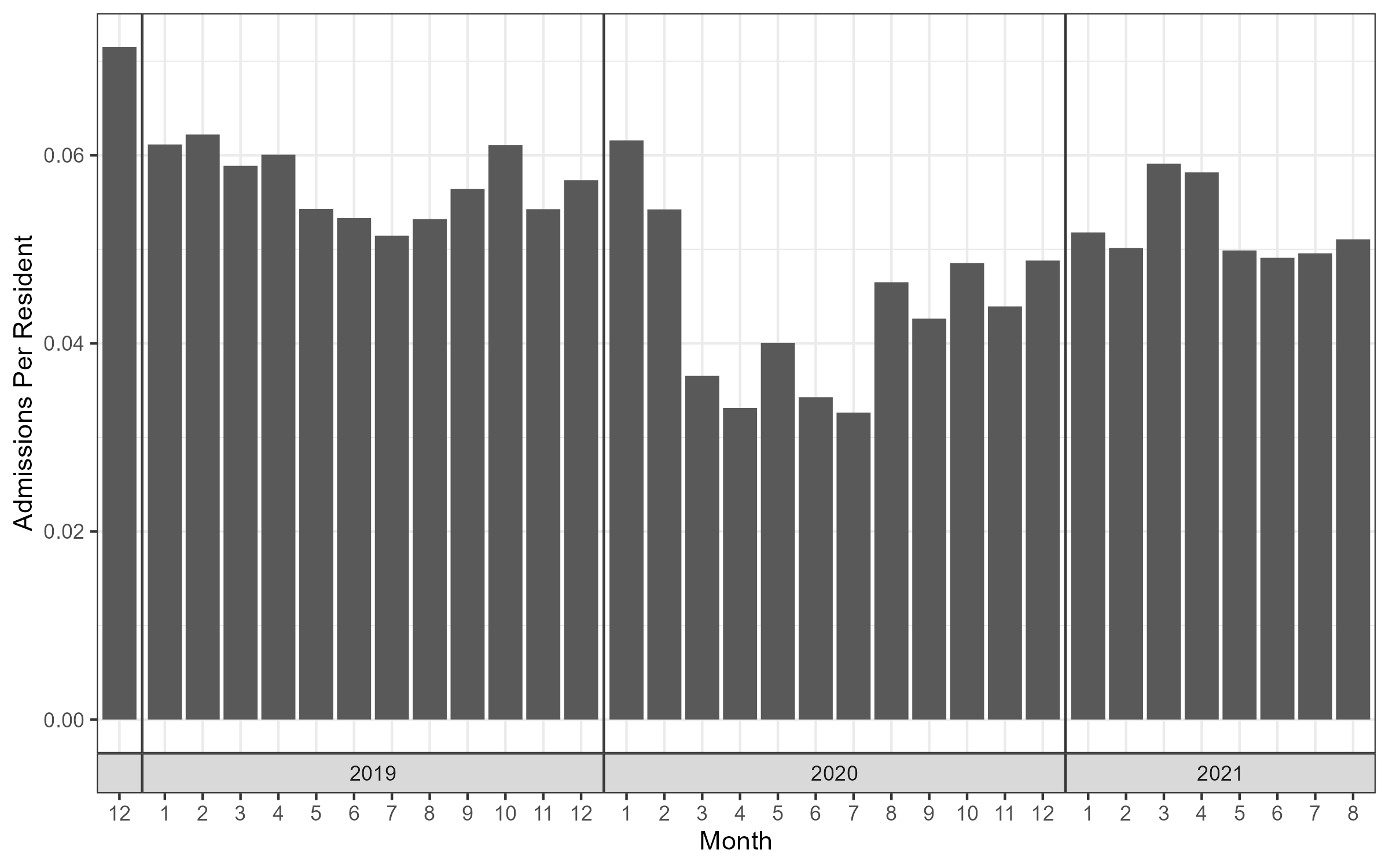


Figure S 9: Number of emergency admissions per resident for each month of the study period.


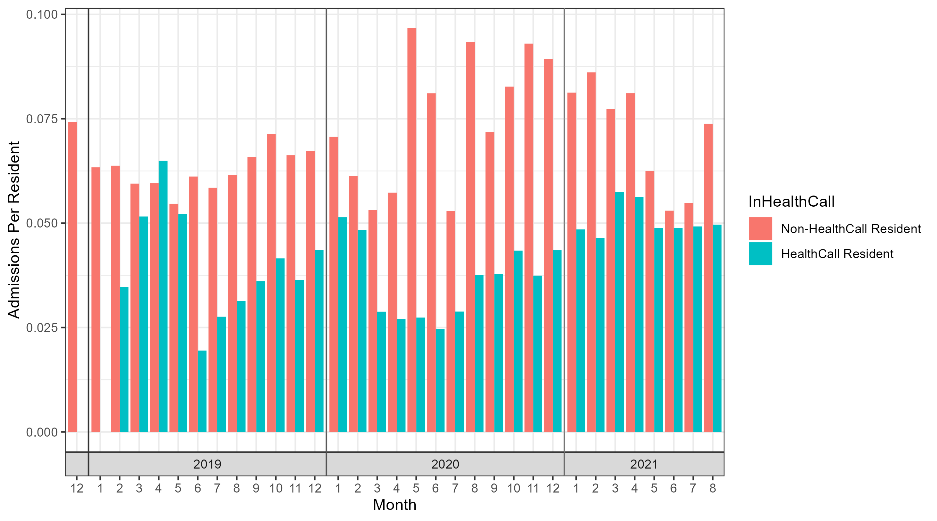


Figure S 10: Number of emergency admissions per resident for each month of the study period, calculated separately for residents on the Health Call system at that point and those who aren’t.

Length of Stay


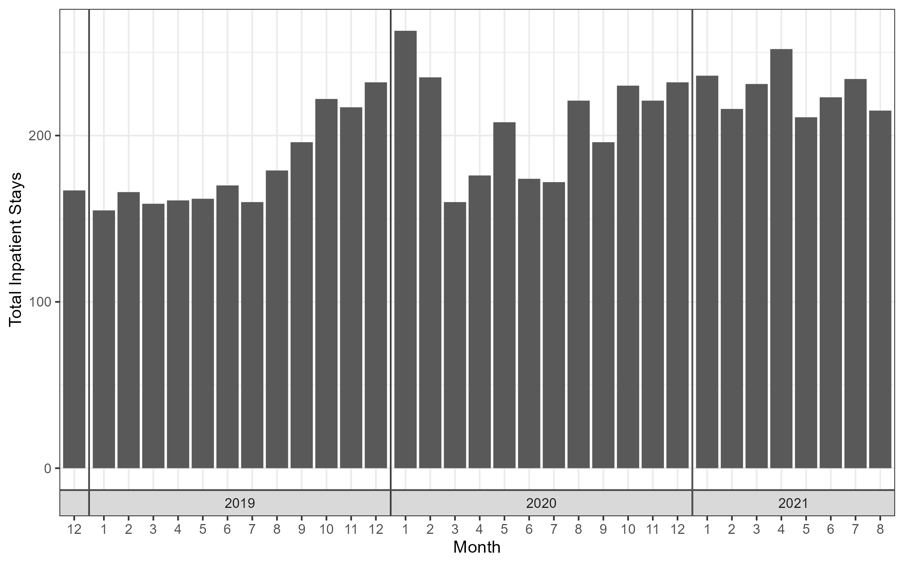


Figure S 11: Total number of emergency inpatient stays each month during the study period for residents in the cohort.


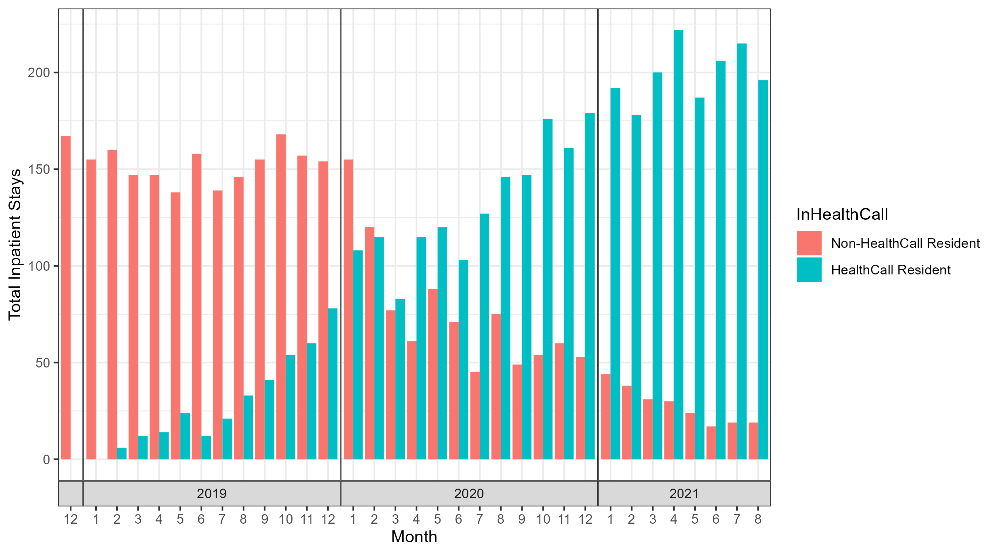


Figure S 12: Total number of emergency inpatient stays each month during the study period for residents in the cohort, separated by residents' Health Call statuses.


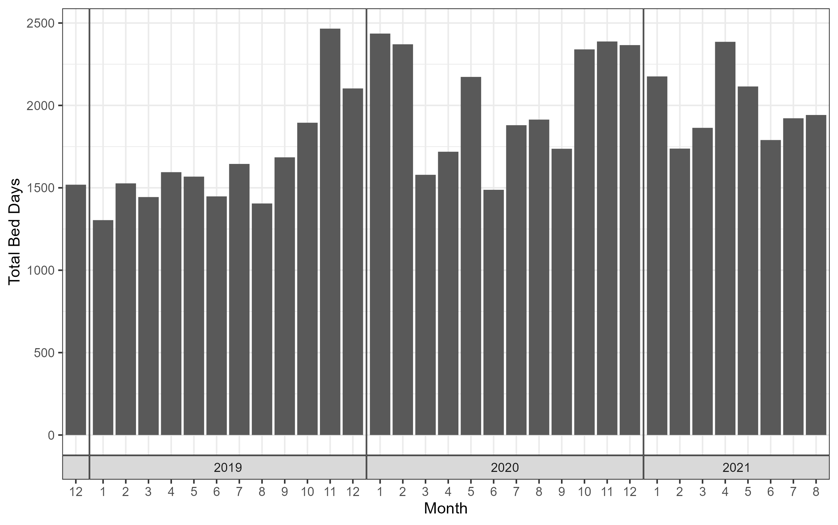


Figure S 13: Total number of bed days for emergency inpatient stays each month during the study period for residents in the cohort.


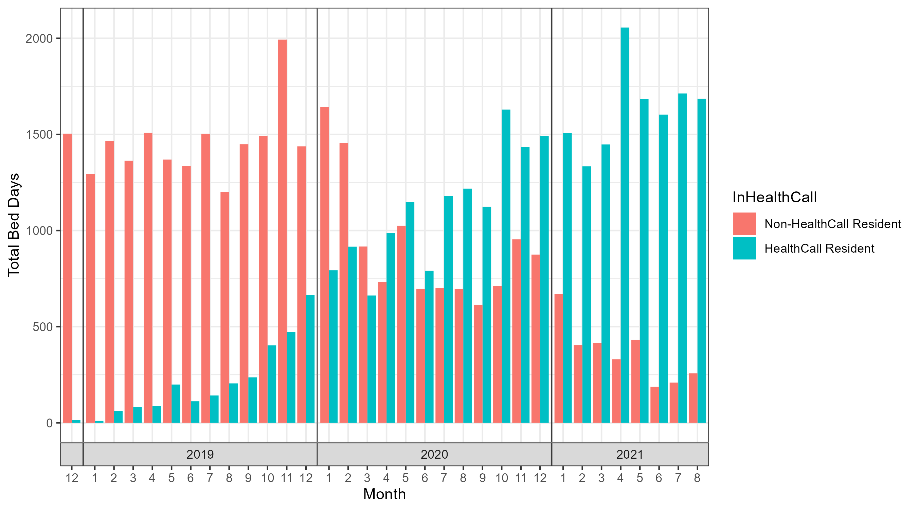


Figure S 14: Total number of emergency inpatient bed days each month during the study period for residents in the cohort, separated by residents' Health Call statuses.


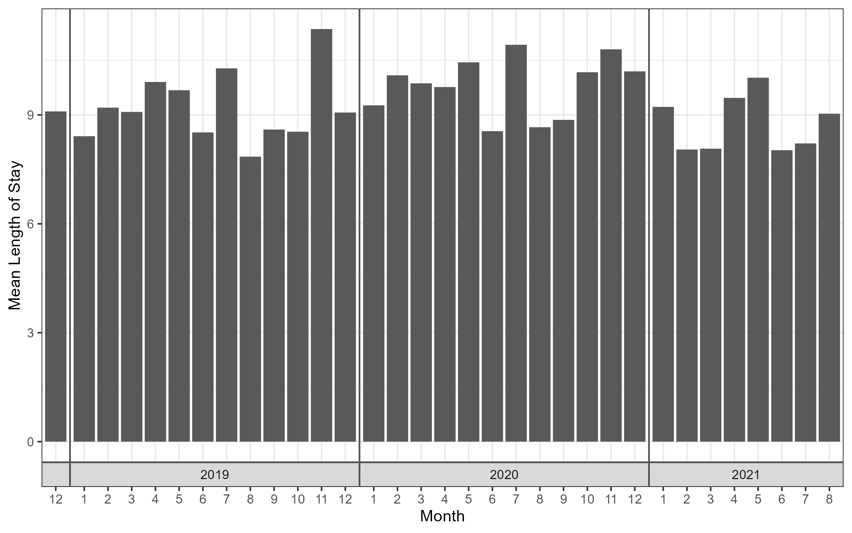


Figure S 15: Mean length of stay for emergency inpatient stays each month for residents in the cohort.


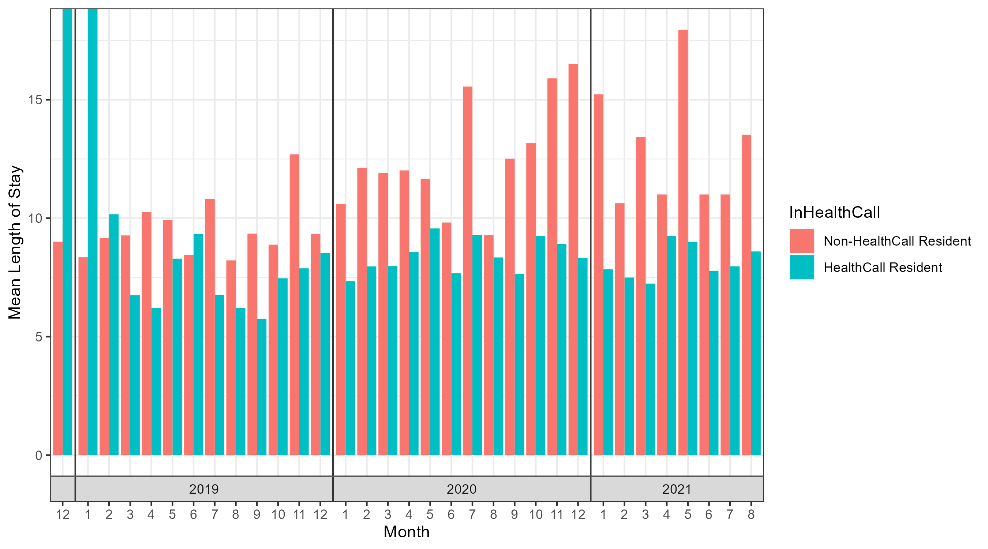


Figure S 16: Mean length of stay of emergency inpatient stays each month during the study period for residents in the cohort, calculated seperately for Health Call residents and non-Health Call residents.

### Additional Economic Analysis Information

Table S 3: Unit costs and sources

| Item of resource | Unit cost  (£, 2019/20) | Source |
| --- | --- | --- |
| District nurse face-to-face visit | 49 | PSSRU, 2020 |
| District nurse other visit | 25 | PSSRU, 2020 |
| Community matron face-to-face visit | 59 | PSSRU, 2020 |
| Community matron other visit | 30 | PSSRU, 2020 |
| Ambulance conveyance to emergency department | 292 | NRC, 2019/20 |
| Emergency department attendance | Various | NRC, 2019/20 |
| Outpatient attendance | Various | NRC, 2019/20 |
| Inpatient cost per day (non-elective) | Various | NRC, 2017/18 |

Table S 4: Predicted monthly costs per resident by calendar year

|  | No Health Call | Health Call | Difference |
| --- | --- | --- | --- |
| 2018 | 322.96 | 265.95 | 57.01 |
| 2019 | 325.30 | 253.93 | 71.37 |
| 2020 | 284.51 | 197.24 | 87.27 |
| 2021 | 287.48 | 174.52 | 112.96 |


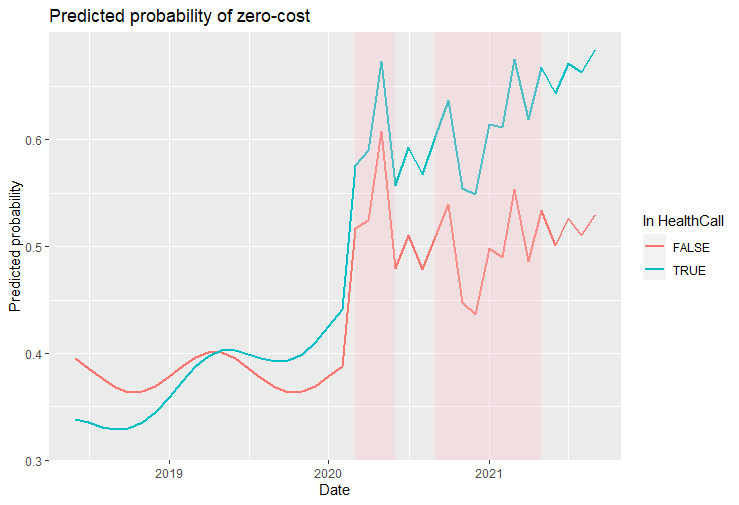


Figure S 17: Predicted probability of resident having zero cost

Health Call is associated with an immediate reduction in the probability of a resident having a zero cost relative to non-Health Call homes, as illustrated by the lower line in 2018 (OR=0.730, p<0.001). However, the probability of a Health Call resident having a zero cost increases monthly relative to non-Health Call residents (OR=1.026, p<0.001). Consequently, the probability of a resident having zero costs becomes greater in Health Call homes.


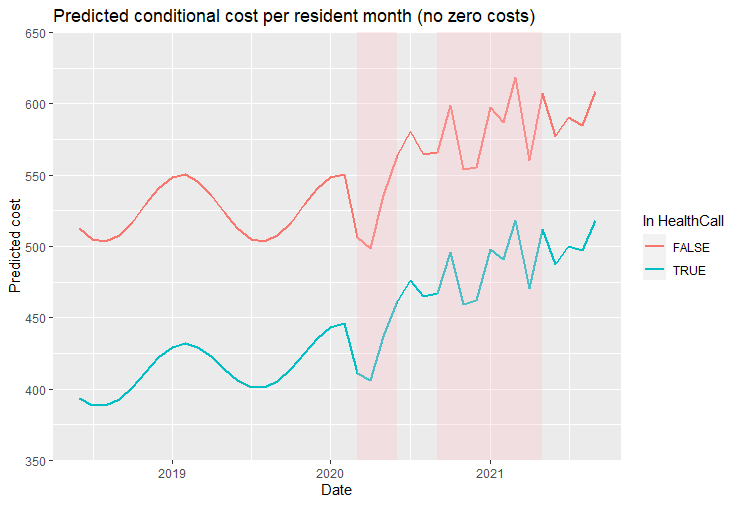


Figure S 18: Predicted cost of residents with non-zero costs

Health Call is associated with an immediate reduction in the predicted costs of residents with non-zero health care costs, relative to non-Health Call residents (PR=0.762, p<0.001). This difference reduces marginally, over time (PR=1.003, p=0.024).
